# The seroepidemiology of Immunoglobulin G antibodies against pertussis toxin in China: a cross sectional study

**DOI:** 10.1186/1471-2334-12-138

**Published:** 2012-06-20

**Authors:** Qi Zhang, Huizhen Zheng, Meizhen Liu, Ke Han, Jun Shu, Chenggang Wu, Ning Xu, Qiushui He, Huiming Luo

**Affiliations:** 1 National Institute for Communicable Disease Control and Prevention, State Key Laboratory for Infectious Diseases Prevention and Control, Chinese Center for Disease Control and Prevention, Beijing 102206, People's Republic of China; 2 Center for Disease Control and Prevention of Guangdong Province, Guangzhou 501300, People's Republic of China; 3 Department of Infectious Disease Surveillance and Control, National Institute for Health and Welfare (THL), Turku and Department of Pediatrics, Turku University Hospital, Turku 20520, Finland; 4 National Immunization Programme, Chinese Center for Disease Control and Prevention, Beijing 100050, People's Republic of China

**Keywords:** Pertussis, Cross-sectional study, Sero-epidemiology, Anti-PT IgG, China

## Abstract

**Background:**

Pertussis is a reported vaccine-preventable respiratory disease in China. Because the routine laboratory methods for diagnosis are not in use, the reported cases are mainly in infants with classical paroxysmal cough and the true incidence related to pertussis is most likely under estimated. In China, however, few studies have attempted to address this issue. The purpose of this cross sectional study was to estimate the incidence rates using the method of sero-epidemiology of immunoglobulin (Ig) G antibodies against pertussis toxin (PT) among healthy populations in China.

**Methods:**

Blood samples were obtained from 1313 healthy individuals aged 0 to 95 years in Guangdong province of China throughout September 2010. Serum IgG antibodies against PT were determined by commercial ELISA kits. Subjects with concentration of anti-PT IgG higher than 30 IU/mL were indicated to have recent *Bordetella pertussis* infection, if they have not received a booster dose of pertussis vaccine within one year.

**Results:**

Of the 1313 study subjects, 117 (8.91%) were found to have anti-PT antibodies higher than 30 IU/mL. The estimated incidence of recent infection was thus 9395 per 100,000 for individuals older than 7 years. Peaks of the estimated incidence rate of recent infection were found to be 11561 per 100,000 in age group of 41–50 years and 11428 per 100,000 in the group aged 13–19 years.

**Conclusions:**

Our study indicated that *B.pertussis* infections are considerablely common, particularly in adolescents and adults in China. The study also stresses the importance of laboratory diagnosis for pertussis and employment of booster dose of pertussis vaccine in adolescents and adults in this country.

## Background

Pertussis, mainly caused by *Bordetella pertussis* is a very communicable disease and primarily affects infants and younger children. Although the disease has been well controlled worldwide since the routine childhood vaccination began in the 1950s, many studies have reported re-emergence of pertussis in European countries and the United States since 1990s. Because of increased circulation of *B. pertussis* and waning vaccine-induced immunity among adults and adolescents, they are the significant source of infection to neonates and younger infants [1]. Studies suggested that there were approximately 48.5 million annual cases of pertussis worldwide, with 295,000 deaths [2,3].

In China, use of whole cell pertussis vaccine combined with diphtheria and tetanus toxoids (DTwP) was started in 1980s. Since 2007, a combined diphtheria-tetanus-acelluar pertussis vaccine (DTaP) has been introduced. Both DTwP and DTaP vaccines are now in use and administered in the 3^th^, 4^th^ and 5^th^ months of life. A booster dose with DTwP or DTaP is given to children aged 18–24 months. According to China official country estimates, the immunization coverage rates and number of districts achieved with 3 doses of DTP vaccination in childhood have been more than 90% since 2002 [4]. The immunization coverage of four doses was over 99% in the year of 2011.

Guangdong province is located in Southern part of China and is considered one of the most economic developed regions in this country. There are 21 prefectural-Level cities with total 104.3 million inhabitants. The vaccination program used in the Guangdong province is the same as the above-mentioned national program. Since 2010 only DTap vaccine is used for pertussis immunizations. The immunization coverage rate has been more than 95% since 1999 in province.

Pertussis is a reportable infectious disease and the number of reported cases has been decreasing in China. Pertussis is clinically diagnosed, and laboratory methods such as serology of ELISA, PCR and culture are not routinely used. Since the 1990s, notified incidence has been less than 1 case per 100,000 population [5,6]. From 2004 to 2011, incidence of pertussis by notification decreased from 0.36 per 100,000 to 0.18 per 100,000. The death rate due to pertussis was less than 0.2%. [7]. Of the 22,571 cases reported during the period of 2004–2011, almost 8,533 (37.8%) were infants. Moreover, a larger proportion of reported cases occurred among children who migrated from rural areas to urban areas with their families. In 2009, only 1,616 cases were reported in China and 17 cases were reported in Guangdong province [8]. Because adults and adolescents often have atypical “whooping cough” symptoms and do not usually seek physicians, the true incidence of pertussis is most likely underestimated[9].

Pertussis toxin (PT) is the most specific antigen for pertussis and cross-reacting antigens have not been described [10]. All of licensed DTaP vaccines contain purified PT. Therefore, IgG antibodies against PT are either a specific indicator of recent pertussis infection in general population [11] or one of indicators for surveillance of the effectiveness of the DTaP vaccines in vaccinated population.

In this study, we wanted to determine concentrations of IgG antibodies to PT among healthy population in Guangdong province, in order to gain an insight into seroepidemiology of pertussis in China, incidence of pertussis infection estimated in adolescents and adults and level of anti-PT IgG antibodies in children (less than 7 years old) vaccinated with DTP vaccines before the life of two years. This study also assessed trends of pertussis and implications for prevention strategies independent of notification and diagnostic bias.

## Methods

### Subjects and study design

Only healthy individuals were enrolled in the study in Guangdong province (with a population of 104.3 million) throughout September 2010. The sample size calculated for this present study was based on the previous results of sero-surveillance of immune response to DTP vaccine and references related to sero-prevalence of pertussis conducted in other provinces [12-16] The calculation of the number of subjects needed in this study was based on the formula n_1_ = Z^2^(1-P)/ε^2^P. For age group of less than 14 years old and over 15 years old, P represented 35% and 15% of pertussis sero-positivity respectively and relative permissible error (ε) was set at 30%. When the ratio of lost follow-up 10% was taken into account, 1.5 times cluster sampling ratio and subjects’ home location evenly distributed in cities and counties, a total of 1138 subjects were needed to be enrolled in this study. In terms of sampling method, all subjects were selected randomly from one county or administrative district affiliated to each of 21 prefectural-Level cities. All subjects were asymptomatic while entering the study. Three individuals with sign of respiratory diseases were excluded. A total of 1323 subjects were recruited. Basic demographic data, for example, age, gender, history of DTP vaccination, address and cough symptom lasting more than one week was recorded at the time of blood sampling. Especially, regarding to the history of DTP vaccination, the vaccination coverage rate of sampling cities was recorded in local Center for Diseases Control and Prevention, and vaccination information for single child subject less than 7 years old was collected from certificates brought by parents or guardians.

### Laboratory methods

Three to five mL of venous blood was drawn. Serum was extracted from the blood samples within 1–2 hours after arrival to local laborotories and stored at −20 °C until transported to the laboratory of Guangdong Provincial CDC using the cold chain system. IgG antibodies against PT were measured quantitatively by a commercial ELISA kit (Virion\serion GmbH, Würzburg, Germany), according to the manufacturer’s protocol. In the ELISA kit, the antibody activities were expressed in International Units/mL(IU/mL) and referred to the “First International Standard for Pertussis Antiserum (Human)”, NIBSC code: 06/140, World Health Organization (WHO). Results of anti-PT IgG were interpreted as positive or negative following the instruction of kit’s manual. Subjects bearing more than 30 IU/mL of IgG against PT were considered seropositive, below 20 IU/mL as seronegative. Combined with the recommendations of previous studies, IgG anti-PT titers ≥ 100 IU/mL detected in a single serum sample were a diagnostic proof of recent or active *B.pertussis* infection, if the subject has not received a booster dose of pertussis vaccine within the previous one year [17,18]. While a concentration of ≥30 IU/mL was considered to indicate a probable recent contact with *B.pertussis*, if no booster has been received within the previous one year.

### Statistical analysis

Data analysis was performed using SPSS 13.0 software (SPSS Inc, Chicago, USA). In order to avoid interference with vaccination induced antibodies, and for estimating the incidence rate of infection among school students (primary school and middle school) and adults, subjects were stratified into nine subgroups according to their age: 0–2 years, 3–4 years, 5–6 years, 7–12 years, 13–19 years, 20–30 years, 31–40 years and 41–50 years, and ≥ 51 years. All subjects were further divided into two crossed groups: living in cities or living in counties. Prevalence of seropositivity of anti-PT IgG and estimated incidence rate of recent *B.pertussis* infection in all subjects and each group were descriptively calculated. Quantitative variables were summarized by means with their 95% Confidence Interval (CI), and qualitative variables were expressed as percentages. The chi-squared test and K independent samples test for qualitative variable, and the one-way ANOVA test for quantitative variables were used for comparisons between subgroups. A P value < 0.05 was considered significant. The serological change trends of anti-PT IgG antibodies by 0–6 year ages were demonstrated by scatter figure.

### Ethical considerations

This study was approved by the Institutional Review Board (IRB) of Chinese Center for Disease Control and Prevention. Informed consent was received from all subjects before the blood samples were taken.

## Results

### Study population

Of the 1323 subjects primarily enrolled in the study, 10 were excluded from the analysis due to absence of the demographic data or poor quality of blood samples. Therefore, all following analyses were thus based on the number of 1313 subjects. Of the 1313 subjects, 654 were male and 659 were female (gender ratio 1.14), while 660 were city livers and 653 were county livers (ratio 1.01). All subjects were grouped in nine subgroups: 80 subjects (6.1%) aged 0–2 years, 79 subjects (6.0%) aged 3–4 years, 79 subjects (6.0%) aged 5–6 years, 61 subjects (4.6%) 7–12 years, 175 subjects (13.3%) 13–19 years, 256 subjects (19.5%) 20–30 years, 204 subjects (15.5%) 31–40 years, 173 subjects (13.2%) 41–50 years and 206 subjects (15.7%) ≥51 years (Table 1). The youngest subject was a three months old boy, and the oldest was a 95 years old woman.

**Table 1:** Demographic data and status of anti-PT IgG antibodies in studied subjects

**Subgroup**	**n**	**Mean of anti-PT IgG and 95% CI (IU/mL)**	**P**^**a**^	**Number of subjects (%) in concentrations of anti-PT IgG**			**P**^**c**^
				**<30IU/mL**	**≥30IU/mL**	**≥100IU/mL**	
Total	1313	14.21(12.29-16.13)		1196(91.09%)	93(7.08%)	24(1.83%)	
Home location							
City	660	14.51(11.43-17.58)	0.76	609(92.27%)	51(7.73%)	13(1.97%)	0.18
County	653	13.92(11.62-16.23)		587(89.89%)	66(10.11%)	11(1.68%)	
Gender							
Male	654	15.09(12.43-17.73)	0.37	589(90.06%)	65(9.94%)	15(2.29%)	0.14
Female	659	13.35(10.57-16.12)		607(92.11%)	52(7.89%)	9(1.36%)	
Age (average age)							
0-2(1.94)	80	10.91(7.01-14.82)	0.55	72(90%)	8(10.0%)	1(1.25%)	0.44
3-4(4.2)	79	9.95(5.74-14.17)		75(94.94%)	4(5.06%)	1(1.26%)	
5-6(5.8)	79	10.80(4.95-16.65)		75(94.94%)	4(5.06%)	1(1.26%)	
7-12(9.4)	61	9.45(6.04-12.86)	0.03^b^	57(93.44%)	4(6.56%)	0(0)	
13-19(17.4)	175	18.44(11.08-25.81)		155(88.57%)	20(11.43%)	6(3.43%)	
20-30(23.8)	256	15.70(9.89-21.5)		237(92.58%)	19(7.42%)	6(2.34%)	
31-40(35.7)	204	13.60(9.85-17.34)		188(92.16%)	16(7.84%)	3(1.47%)	
41-50(46.0)	173	15.42(9.34-21.51)		153(88.44%)	20(11.56%)	3(1.73%)	
> 51(64.5)	206	14.02(10.94-17.10)		184(89.32%)	22(10.68%)	3(1.46%)	

^a^ Based on two sample *t*-test and one-Way ANOVA.

^b^ Two sample *t*-test between 1–2 years and 13–19 years group.

^c^ Based on nonparametric tests for 2 independent sample and Kruskal-wallis H.

### Concentrations of IgG antibodies against pertussis toxin

The mean concentration of anti-PT IgG antibodies in 1313 study subjects was 14.21 IU/mL (95%CI: 12.29-16.13) (Table 1). Low levels of anti-PT antibodies were observed in all age groups. The mean levels of anti-PT IgG and 95% CI of each age group were listed in Table 1. The highest mean concentration of anti-PT IgG was found in 13–19 years group with 18.44 IU/mL (95% CI 11.08-25.81). The lowest level was 9.95 IU/mL in group of 3–4 years age. No difference in the mean concentration of anti-PT IgG was found between subjects living in cities and counties and between the genders (P = 0.76 and 0.37). Although no difference in the mean concentration of anti-PT IgG antibodies was observed among nine subgroups by using one-way ANOVA test (P = 0.55), the mean concentration of anti-PT IgG in subjects aged 13–19 years was significantly higher than those aged 7–12 years analyzing by two sample *T*-test (P = 0.03).

### Sero-prevalence of IgG antibodies against pertussis toxin among populations aged more than 7 years

Of the 1075 subjects older than 7 years of age, 101 (9.39%, 95%CI 7.65%-11.13%) had anti-PT IgG antibodies higher than 30 IU/mL and were thus considered seropositive for *B.pertussis*, and 21 (1.95%, 95%CI 1.12%-2.78%) had concentration of anti-PT IgG antibodies higher than 100 IU/mL (Table 1). The subjects aged 13–19 years had highest sero-positivity rate 3.43% (95% CI 0.73%-6.13%) in ≥100 IU/mL category, and the subjects of 41–50 years age had highest sero-positive rate 11.56% (95% CI 6.80%-16.32%) in ≥ 30 IU/mL. The number of study subjects included in the age group of 7 to 12 years was relatively low,and the lowest positivity rate 6.56% was observed (Table 1). When the sero-positivity rates were compared among age subgroups, no statistically significant difference was found by using Kruskal-Wallis Test (P = 0.437). The sero-positivity rate of all age groups was shown in Table 1.

### Sero-prevalence of anti-PT antibodies among children younger than 7 years of age

**Figure 1:**
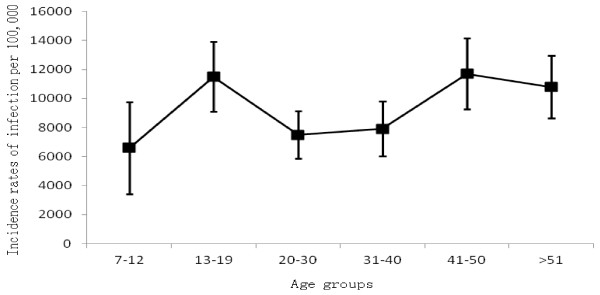
** Concentrations of anti-PT IgG antibodies in 238 healthy children aged less than 7 years.** Each symbol represents one subject. Solid line presents mean level of IgG using local regression analysis.

The concentrations of anti-PT IgG antibodies of 238 children less than 7 years old were plotted in Figure 1. Most of children had anti-PT antibodies below 30 IU/mL. Only three children were found to have anti-PT IgG antibodies higher than 100 IU/mL. However, the guardians of the three children didn’t claim that their children suffered cough at the time of blood sampling. All children studied had received DTaP or DTwP vaccines on time according to the vaccination certificates. A 3-month-old infant had just received first dose of DTaP vaccine and the anti-PT IgG tested was 52.54 IU/mL. A 4-month-old infant had received 2 doses of DTaP vaccine and the concentration of anti-PT IgG antibodies was only 2.16 IU/mL. Moreover, the serological trends of anti-PT IgG level among children (over 6 months old) who received 3 doses DTP were as similar as that of children (over 2 two years old) who received booster dose DTP, and the trend was also no distinctive change till 7 years old children.

### The estimated incidence rates of *B.pertussis* infection

**Figure 2:**
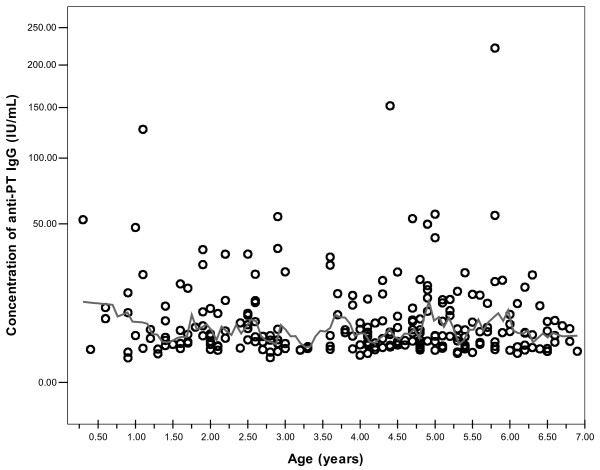
** The age-sepcific estimated incidence rates of *B.pertussis* infection per 100,000 in the year before serum sampling based on the cut-off 30IU/mL.**

Depending on the pertussis vaccination programs, various cutoff values for diagnostic anti-PT IgG antibodies have been proposed for adolescents and adults [18]. However, there are no available cutoff values of anti-PT IgG as indication as *B.pertussis* infection used in China. In this study, we considered the mean level of anti-PT IgG of all subjects and the instruction of ELISA kit for results judgment and previous studies on the decay of anti-PT IgG titer after infection [17,19] and decided to apply 30 IU/mL in estimating the incidence rate of *B. pertussis* infection. Moreover, the concentration of anti-PT antibodies expressed as IU/ml in our present study was equivalent to that expressed as U/ml used in an earlier study carried out in the Netherlands by de Melker’s study [17]. In the Dutch study, it was found that after infection, anti-PT IgG titers take on average 58.6 days to drop to a level of 100 IU/mL and 365 days (one year) to reach a level of 30 IU/mL, respectively. Based on 9.39% of subjects more than 7 years age fell in the category of ≥30 IU/mL, an estimated incidence rate of probable *B.pertussis* infection in the year before the serum sampling is 9.39% (365.25 days/365 days × 9.39%). In Figure 2, the age-specific incidence rates of infection with *B. pertussis* in individuals more than 7 years of age are given as calculated for the cut-off ≥30 IU/mL. The age distribution of estimated infection rates also revealed that the peak of estimated incidence rate of recent infection was in the age catergory of 41–50 years (11561/100,000), and the second peak of the estimated recent infection rate was in the group of 13–19 years age (11428/100,000). The estimated incidence rate of the adolescence and adults produced ten thousand times higher than that reported in China.

## Discussion

In China, 90% reported pertussis cases were infants or young children who suffered from classical “whooping cough” symptoms. Studies suggested that only 40-50% of pertussis cases had a classical clinical manifestation of a paroxysmal cough [20], often leading to a misdiagnosis as a general respiratory infection and a failure to investigate for pertussis. Since the methods for laboratory confirmation of *B.pertussis* were not routinely used in China, many of pertussis cases without classical paroxysmal cough symptom among vaccinated children, adolescents and adults failed to be notified and were possibly considered as common respiratory infections. The misdiagnoses clearly contributed to the reported low incidence under the 1 per 100,000 since 1990s in China. In Guangdong, the number of reported pertussis cases ranged from 18 to 115 cases in each year during the period of 2004–2011, and only one death was reported in 2005. There were only 18 and 22 cases notified in 2010 and 2009. Therefore this cross sectional study was aimed to investigate seroprevalence of pertussis in all age groups in a region of China.

Threshold employed in this study were based on combination of mean value of anti-PT IgG of studied population, China pertussis vaccine programs, instruction of ELISA kit and previous studies of EU. Mean concentration of anti-PT IgG in subgroups of subjects was relatively low fluctuating between 10 IU/mL and 20 IU/mL. This result was similar to our recent study carried out in Shandong province, eastern part of China [21]. In China, no booster dose was used in adolescents and/or adults. The levels of anti-PT IgG antibodies observed in China might be lower than that of European countries and US in which boosters for adolescents and young adults were introduced [22,23]. Regarding the performance of available commercial ELISA kits for diagnosis of pertussis, a recent German study showed that only kits using PT as a coating antigen gave overall good sensitivities and specificities compared to their in-house ELISA [18], suggesting that our results obtained by using the commercial ELISA kits are true. Therefore, considering the high specificity of anti-PT IgG, one cut-off was set at 30 IU/mL rather than a higher cut-off with lower sensitivity, which was used to indicate a probable recent contact with *B.pertussis,* if no booster has been received within the previous one year. Various cutoff values for anti-PT IgG with recent contact to *B. pertussis* antigens have been proposed by various countries [19,24-26]. In this study, we also applied the diagnostic algorithm described by Riffelmann that anti-PT IgG ≥100 IU/mL as diagnostic of recent or active infection with *B.pertussis*.

In order to avoid the possible influence of antibodies derived from vaccinations, we mainly estimated the incidence rates among individuals older than 7 years age based on high anti-PT IgG titer in serum sample. It is known that the vaccine-induced antibodies began to wane 3 to 5 years after the last dose of vaccination, and immunity to pertussis vaccine diminished to 0%-20% over a 10-year interval [27,28]. Primary pertussis vaccination in China has been targeted routinely only at the infant age group with a fourth dose injected at 18–24 months, and no further booster doses were used in this country. High level of anti-PT IgG antibodies in subjects older than 7 years age are most likely due to contact of *B.pertussis.* Therefore, information about the sero-prevalence of high levels of anti-PT antibodies in combination with the post-infection antibody decline rate allows us to study *B.pertussis* infections in > 7 years age groups irrespective of clinical manifestation and reporting symptom.

Our results clearly showed that despite a high vaccination coverage (>98%) in Guangdong province, pertussis is common in the communities, particularly in the adolescents and adults. The estimated incidence rate of recent infection was found that 9.39% (or 9395 per 100,000) of the population older than 7 years of age had experienced contact with *B. pertussis* in the year before sampling, which was found to be ten thousand times higher compared to that based on the notifications in 2010 (0.176/100,000). Many countries have observed pertussis is under-reported and under-diagnosed [29], for example, the estimated incidence rates of infection is more than 600-times higher than the notified cases numbers in Netherland [17]. We have to realize that raised anti-PT IgG antibodies could reflect exposure/infection rather than clinical disease. Due to absence of clinical symptom, many adults cases with higher anti-PT IgG were considered as atypical or asymptomatic infection. The high proportion of study subjects with atypical or asymptomatic infection clearly contributed to the high estimated incidence rate of infection observed in this study. It remains to be shown if the pathogen of *B. pertussis* could be transmitted by subjects with asymptomatic or atypical infections.

Previous studies suggest that the amount of under-reporting in pertussis varies among different age groups, and the under reporting was higher for older children, adolescents, and adults than for younger children [30,31]. Our results were in consistent with the earlier findings. In this study, the higher incidences were observed in adolescents and adults older than 40 years of age. Due to more crowding students in schools, high rate of person to person transmission in this age group can occur and therefore the incidence rate of recent infection was highest. In addition, our data also revealed the relative higher sero-positive among population living in county than in city. According to the reported vaccination coverage rate in Guangdong province, the lower access to health facilities didn’t seem to produce lower immunization coverage. One explanation might be that due to the low number of hospitals or department of diseases control and prevention and lack of awareness to pertussis, subjects with pertussis could not be diagnosed or could not have access to treatment and control strategy. This may have resulted in disease transmission.

The concentrations of anti-PT antibodies induced by vaccination or by infection couldn’t be differentiated by laboratory technique, eg, ELISA, but in this study, a distinctively increased level of anti-PT IgG was not noted after fresh vaccination among 0–2 years group. In China, children receive four doses of pertussis vaccines before two years of age. Therefore, higher level of anti-PT antibodies in age group 0–2 compared to other age groups would have been expected. In this present study, however, we did not observe such a difference. One explanation might be that homemade DTwP or DTaP vaccines from various manufactuers differ considerably in their immunogenicity. According to the Chinese Pharmacopoeia, DTaP are mainly composed of PT and FHA in pertussis ingrendients, but the proportion and content of two antigens were not described in instruction. It was indicated in other study that apart from high anti-PT antibody titers as correlate of protection, high titer of anti-FHA Antibody can also provide some protection against pertussis [32]. This could be one explanation. In this study we didn’t measure anti-FHA Abs among children who received homemade DTP vaccines. However we believed that the increasing immunization coverage in China should contribute to the decreasing trend in pertussis cases reported. Unfortunately we do not have efficacy data from the vaccine trials in China. Another explanation might be the low number of studied subjects included in the age group.

We had to realize that the serological cut-off value for estimating the incidence was consulted to the data of present study on asymptomatic population and previous studies of EU. We have not validated its power for diagnosing pertussis patients who were confirmed by other methods such as PCR or culture. A prospective cohort study on pertussis infection in hospitalization is being planned in China CDC in which all three laboratory methods PCR, culture and serology ELISA will be used. Since China is a big country with 1.3 billion populations and this study was only carried out in one province, a multi-center studies should be conducted to further understand the burden of pertussis in this country.

## Conclusion

In conclusion, our study indicated that in China *B.pertussis* infections are considerablely common, particularly in adolescents and adults. This study also stresses the importance of laboratory diagnosis for pertussis and employment of booster dose of pertussis vaccine in adolescents and adults in China.

## Competing interests

The authors declare that they have no competing interests.

## Authors’ contributions

Q Zhang, HZ Zheng and HM Luo planned the study. CG Wu, K Han, J Su and N Xu were in charge of data collection and blood samples collection. MZ Liu carried out the immunoassays. Q Zhang performed the statistical analysis. Q Zhang drafted and edited the manuscript. Q He participated in data analysis and edited the manuscript. All authors read and approved the final manuscript.

## Pre-publication history

The pre-publication history for this paper can be accessed here:

http://www.biomedcentral.com/1471-2334/12/138/prepub
